# Aponeurotic Expansion as a Cause for Rotator Cuff Tears but Full Joint Movements in Patients: Magnetic Resonance Arthrography Images of Aponeurotic Expansion and the Clinical Situation

**DOI:** 10.7759/cureus.66272

**Published:** 2024-08-06

**Authors:** Veysel Uludağ, Derya Güçlü, Zekeriya Okan Karaduman, Hayri Ogul

**Affiliations:** 1 Physical Therapy, Duzce University, Duzce, TUR; 2 Radiology, Duzce University, Duzce, TUR; 3 Orthopaedics and Traumatology, Duzce University, Duzce, TUR

**Keywords:** mr arthrography, shoulder function, supraspinatus tendon, aponeurotic expansion, rotator cuff tear

## Abstract

This case report presents the detailed clinical and radiological findings of a 63-year-old male patient who presented with right shoulder pain. Magnetic resonance imaging (MRI) and magnetic resonance arthrography (MRA) revealed significant tears in most of the rotator cuff muscles. Despite these findings, the patient was able to perform full shoulder movements, suggesting that aponeurotic expansion may play a crucial role in this scenario. This case highlights important clinical findings that could lead to potential changes in shoulder surgery and rehabilitation approaches.

## Introduction

The shoulder joint is one of the most mobile joints in the human body, allowing for a wide range of motion. It is stabilized by various muscles, tendons, and ligaments that connect the humeral head to the glenoid fossa. The rotator cuff muscles are particularly important for maintaining shoulder stability and enhancing shoulder range of motion. Tears in the rotator cuff muscles or tendons typically result in significant functional loss and pain [1, 2]. However, in some cases, patients can maintain normal joint movement despite having substantial muscle tears. This phenomenon may be related to aponeurotic expansion.

Aponeuroses are connective tissue structures that transition muscle force to tendons, playing a vital role in distributing muscle force. Aponeurotic expansion may serve as a mechanism to mitigate the effects of muscle tears by distributing forces more effectively. Moser et al. investigated the anatomy and prevalence of aponeurotic expansion in the supraspinatus tendon and highlighted its role in shoulder pathology [3]. Additionally, the functional and mechanical properties of aponeuroses significantly impact the dynamic stability of the muscle-tendon unit [4]. Wheatley et al. studied the microstructural heterogeneity of aponeuroses and its implications for muscle-tendon unit mechanics and suggested that aponeurotic structures play a crucial role in force distribution [5].

Since there are only two anecdotal reports [3, 4] in the literature regarding shoulder aponeurosis and clinical status, we aimed to indicate in this case report that there may be a relationship between shoulder function and expansion of the aponeurosis.

## Case presentation

A 63-year-old male presented to our clinic with a two-month history of right shoulder pain. The patient had worked in the construction industry under heavy physical conditions for 40 years, had no history of smoking or alcohol use, and had never undergone surgery. Clinical examination revealed a nearly full passive and active range of motion in the shoulder joint, with minimal pain reported at the end range of passive movement. The patient's rotator cuff muscle strength was subjectively evaluated as four out of five.

Pain severity was assessed using the visual analog scale (VAS), with a score of 0 at rest and three during activity. The Disabilities of the Arm, Shoulder, and Hand (DASH) score was 70 out of 100, indicating moderate disability. Patient quality of life was assessed using the 36-item Short Form Survey Instrument (SF-36) questionnaire, with scores as follows: physical function 85, social function 90, general health 75, mental health 80, vitality 70, pain 40, emotional role function 60, and physical role function 55.

Shoulder intraarticular injection was performed using a 20-G needle via a posterior approach on an outpatient basis without sedation or premedication using an ultrasonography system equipped with a broadband 7.5-MHz linear transducer. A diluted contrast medium at a concentration of 1:200 was injected. A volume of 15 mL of gadolinium-based solution was injected until the joint capsule was appropriately distended. Magnetic resonance arthrography (MRA) was performed within 10-15 minutes after joint injection using a 3-Tesla MRI (Magnetom Skyra; Siemens Healthcare, Erlangen, Germany). The MRA protocol included turbo spin echo (TSE) T1-weighted MRA in the sagittal plane and fat-suppressed TSE T1-weighted MRA in three planes. Additionally, a fat-suppressed 3D volumetric interpolated breath-hold examination (VIBE) and thin-section coronal plane T2 sequences were added to the MRA protocol.

**Figure 1:**
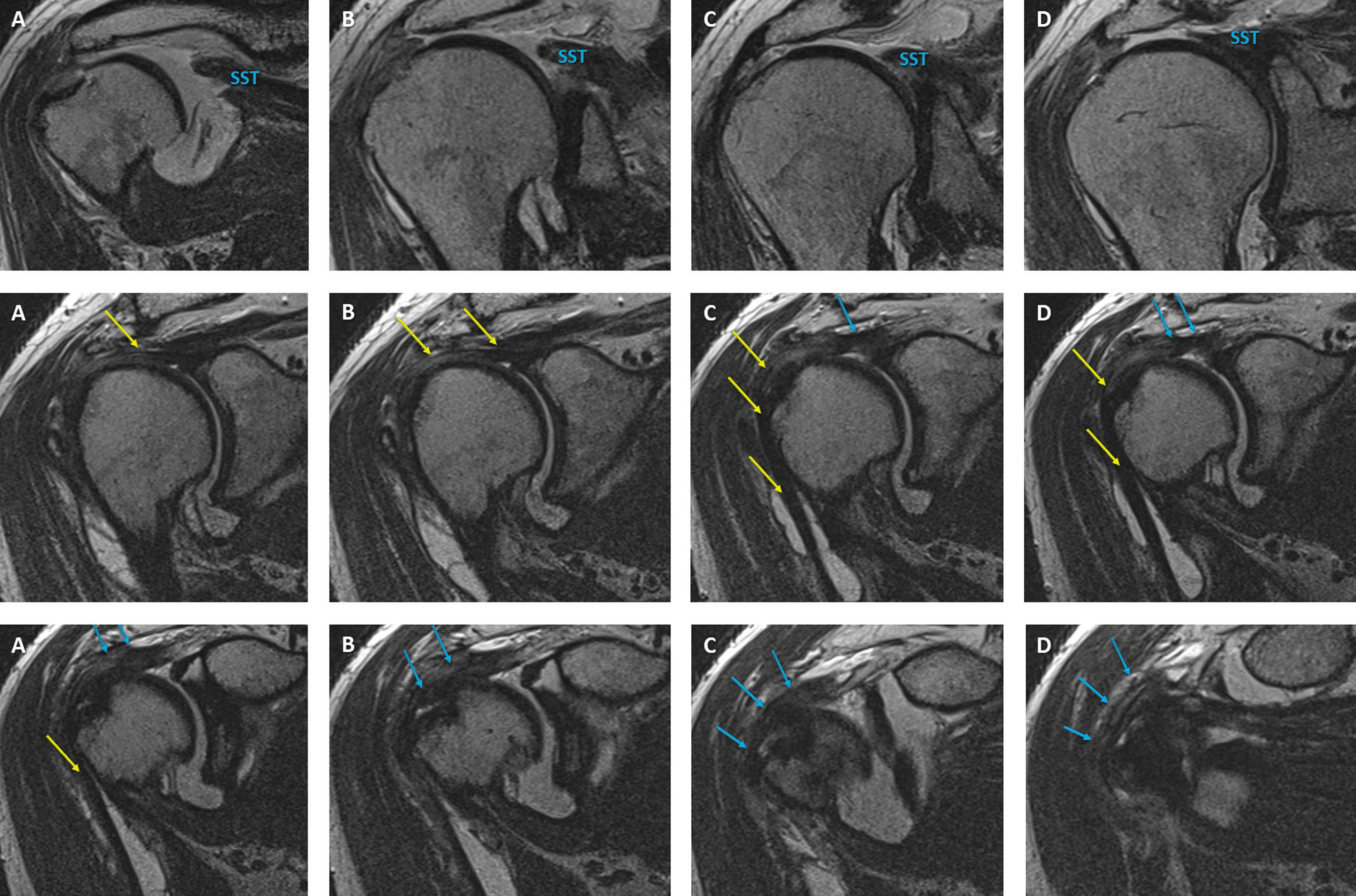
Sequential coronal oblique TSE T2-weighted MR arthrogram sections Consecutive coronal oblique turbo spin echo (TSE) T2-weighted MR arthrogram slices demonstrate complete rupture of the supraspinatus tendon (SST) and aponeurotic expansion of the supraspinatus tendon (blue arrows). Yellow arrows indicate the long head of the biceps brachii tendon.

**Figure 2:**
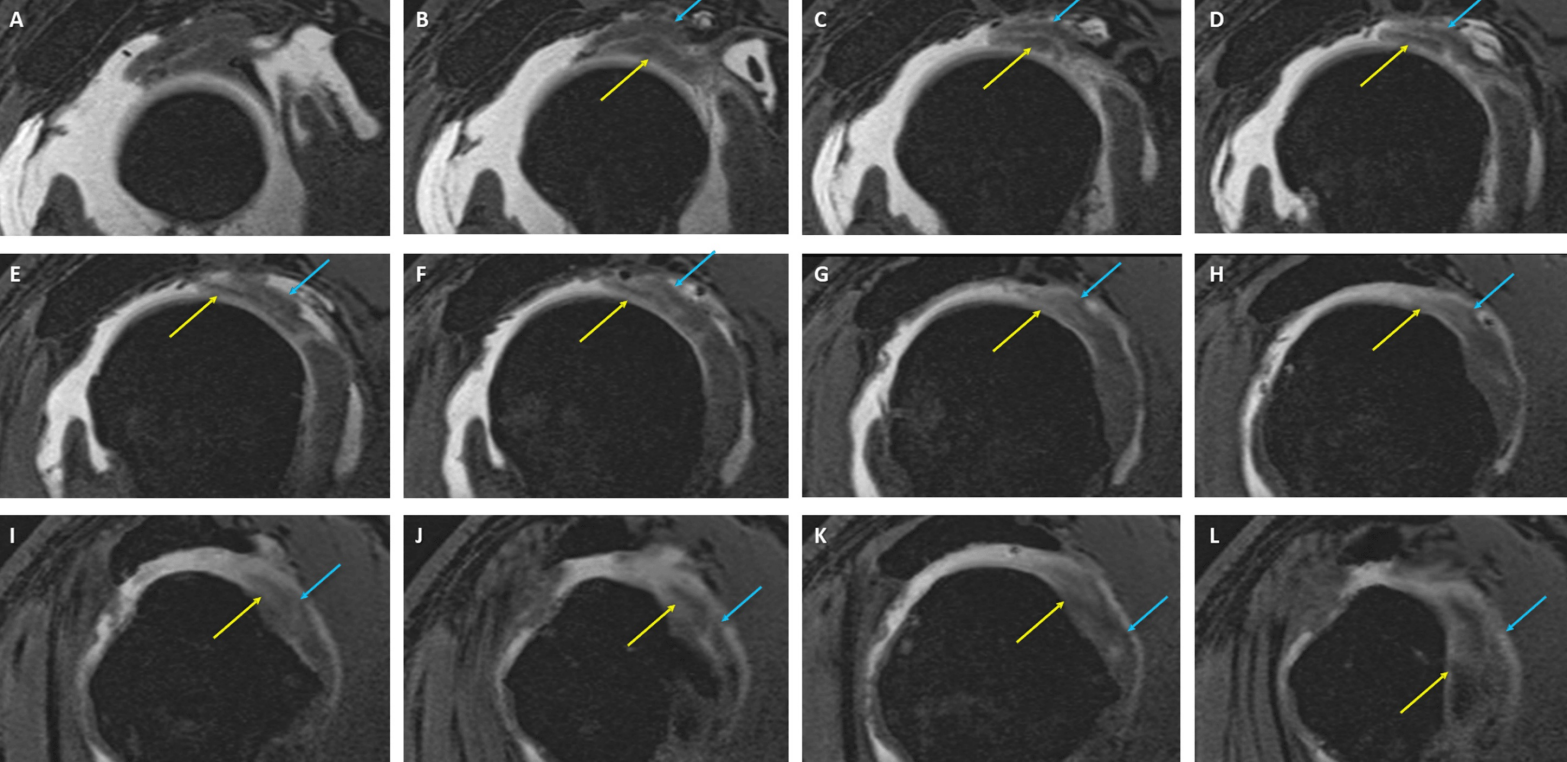
Sequential sagittal oblique T1-weighted VIBE MR arthrogram sections Consecutive sagittal oblique T1-weighted volumetric interpolated breath-hold examination (VIBE) MR arthrogram slices demonstrate nearly complete rupture of the supraspinatus and infraspinatus tendons and aponeurotic expansion of the supraspinatus tendon (blue arrows). Yellow arrows indicate the long head of the biceps brachii tendon.

**Figure 3:**
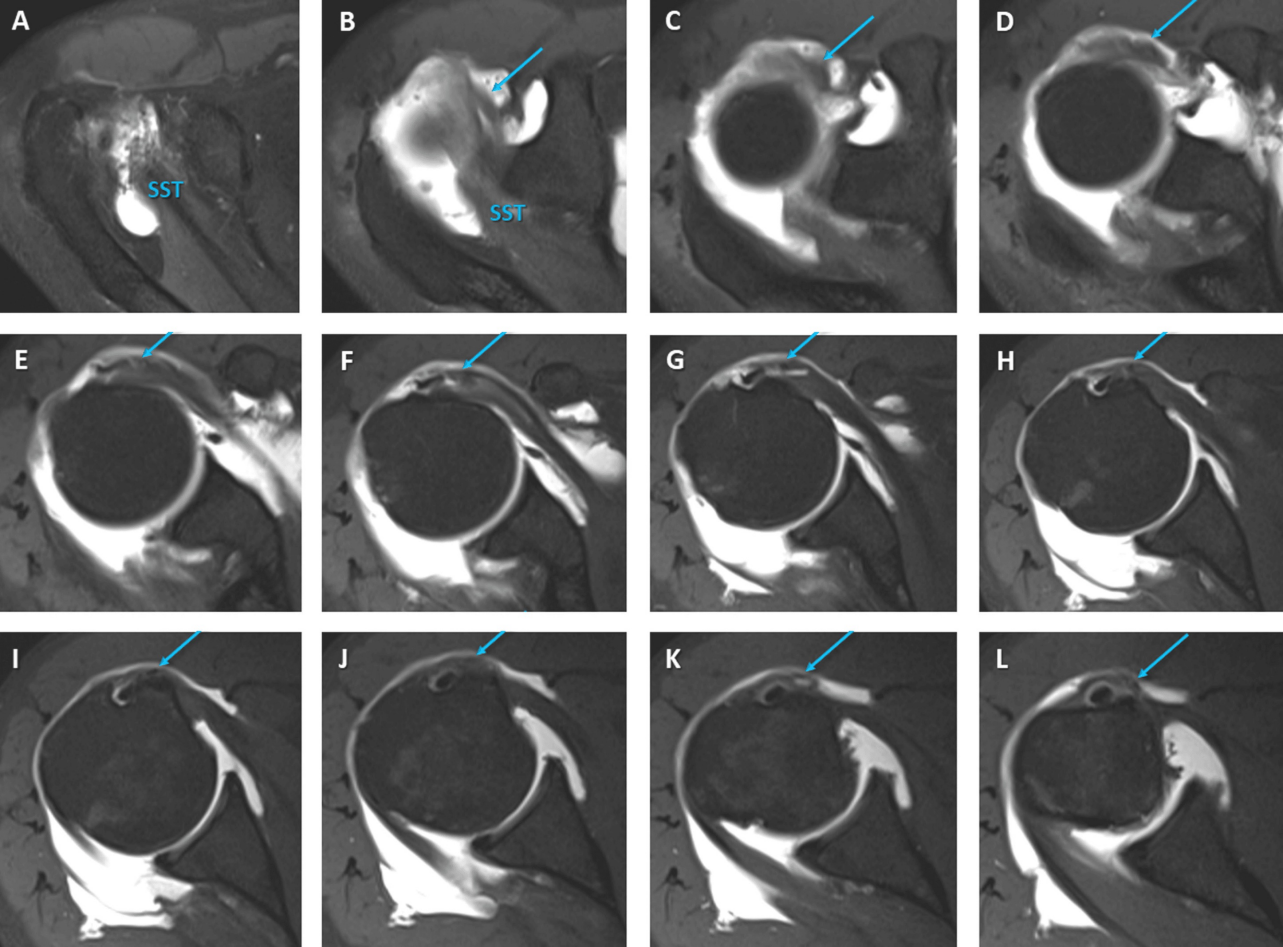
Consecutive transverse plane turbo spin echo (TSE) T1-weighted MR arthrograms It shows almost complete tearing of the supraspinatus (SST) and infraspinatus tendons, partial tearing of the subscapular tendon, and aponeurotic enlargement of the supraspinatus tendon (blue arrows).

Conventional MRI and MRA of the right shoulder revealed a nearly complete rupture of the supraspinatus and infraspinatus tendons and a partial rupture of the subscapularis tendon. Interestingly, MR and MRA images revealed intact aponeurotic expansion of the supraspinatus tendon (Figures 1-3). There was also instability of the long head of the biceps tendon.

The patient was advised to continue with conservative management, including physical therapy focused on strengthening the surrounding musculature. Follow-up after six months showed significant improvement in pain levels and shoulder function, with the patient reporting minimal discomfort and near-normal use of the shoulder in daily activities. The initial complaints were largely resolved, underscoring the potential role of aponeurotic expansion in maintaining joint function despite major tendon tears.

## Discussion

This case underscores the potential role of the aponeurotic expansion of the supraspinatus tendon in massive rotator cuff tendon tears. Aponeurotic structures may help maintain joint function despite significant muscle tears. Our study presents a unique case in the literature, highlighting the presence of intact aponeurotic expansion in a patient with massive rotator cuff tendon rupture, as demonstrated by MRA. Clinically, shoulder joint function was preserved, making this the first documented case of its kind. This finding suggests that the preservation of aponeurotic structures can support shoulder function, which is a significant contribution to the literature.

Akkaya et al. examined the relationship between aponeurotic expansion in the supraspinatus tendon and pathologies of the long head of the biceps and rotator cuff tendons and reported a significant association [3]. Unlike our case, these studies did not explicitly demonstrate the clinical impact of aponeurotic structure preservation. Moser et al. explored the anatomy and prevalence of aponeurotic expansion in the supraspinatus tendon, highlighting its potential role in shoulder pathology [4]. However, these studies did not focus as specifically on the preservation of shoulder function as our case does.

The functional and mechanical properties of aponeurotic structures are crucial for the dynamic stability of the muscle-tendon unit. Wheatley et al. studied the microstructural heterogeneity of aponeuroses and their impact on muscle-tendon unit mechanics and suggested that these structures play a vital role in force distribution [5]. Additionally, Raiteri reviewed the behavior of aponeuroses during muscle contraction, discussing how their variable stiffness can enhance muscle performance and reduce elastic energy consumption [6]. Our study highlights the importance of these mechanical properties in maintaining shoulder joint function.

The evaluation of shoulder pathologies and rotator cuff tears is essential for radiologists and clinicians. Radiological imaging provides detailed insights into the pathology, while clinical assessments determine the patient's functional status and quality of life. The role of aponeurotic structures should not be overlooked in these evaluations. Narvani et al. compared surgical and nonsurgical treatment routes for degenerative rotator cuff tears, highlighting the clinical outcomes of both approaches [7]. Lewis discussed the assessment and management of rotator cuff-related shoulder pain, emphasizing that exercise therapy offers outcomes comparable to those of surgery [8]. Kuhn et al. evaluated the effectiveness of physical therapy in treating atraumatic full-thickness rotator cuff tears and reported that it was effective for most patients [9]. In their study, even though there are several hypotheses for the reason for the improvement of the patients and their response to physical therapy, perhaps the reason for the improvement in these patients may be the enlargement of the aponeurotic structure.

Clinicians often encounter patients with rotator cuff tears who retain a full range of motion and are pain-free. These conditions are often attributed to compensation mechanisms by surrounding muscles, yet there is no clear evidence supporting this claim [10]. The presence of aponeurotic expansion might explain why physical therapy is effective for some patients but not others. Guclu et al. described the 2D and 3D MR arthrographic features of aponeurotic expansion in supraspinatus tendon and biceps tendon anomalies and reported a significant association with shoulder pathologies [11]. This highlights the importance of considering aponeurotic structures in clinical evaluations and treatment approaches. It has been proposed that the expansion of aponeuroses provides substantial biomechanical support. Therefore, collaborative biomechanical studies between clinicians and radiologists are recommended.

## Conclusions

In conclusion, the aponeurotic expansion of the supraspinatus tendon can be preserved even in massive rotator cuff tendon ruptures. This compensating anatomical structure plays a key role in preserving shoulder joint function in such patients. Therefore, we believe that it would be useful for clinicians to develop treatment modalities aimed at aponeurotic expansion in patients with massive rotator cuff tendon ruptures and for radiologists to consider aponeurosis in the diagnosis of shoulder pathologies that cannot be explained by a tear alone. However, there is still a need for more extensive studies on this subject.
